# A Machine Learning-Based Algorithm for Water Network Contamination Source Localization

**DOI:** 10.3390/s20092613

**Published:** 2020-05-03

**Authors:** Luka Grbčić, Ivana Lučin, Lado Kranjčević, Siniša Družeta

**Affiliations:** 1 Department of Fluid Mechanics and Computational Engineering, Faculty of Engineering, University of Rijeka, 51000 Rijeka, Croatia; lgrbcic@riteh.hr (L.G.); ilucin@riteh.hr (I.L.); sinisa.druzeta@riteh.hr (S.D.); 2 Center for Advanced Computing and Modelling, University of Rijeka, 51000 Rijeka, Croatia

**Keywords:** machine learning, artificial neural networks, random forests, water network pollution, sensor networks, parallel computing

## Abstract

In this paper, a novel machine learning based algorithm for water supply pollution source identification is presented built specifically for high performance parallel systems. The algorithm utilizes the combination of Artificial Neural Networks for classification of the pollution source with Random Forests for regression analysis to determine significant variables of a contamination event such as start time, end time and contaminant chemical concentration. The algorithm is based on performing Monte Carlo water quality and hydraulic simulations in parallel, recording data with sensors placed within a water supply network and selecting a most probable pollution source based on a tournament style selection between suspect nodes in a network with mentioned machine learning methods. The novel algorithmic framework is tested on a small (92 nodes) and medium sized (865 nodes) water supply sensor network benchmarks with a set contamination event start time, end time and chemical concentration. Out of the 30 runs, the true source node was the finalist of the algorithm’s tournament style selection for 30/30 runs for the small network, and 29/30 runs for the medium sized network. For all the 30 runs on the small sensor network, the true contamination event scenario start time, end time and chemical concentration was set as 14:20, 20:20 and 813.7 mg/L, respectively. The root mean square errors for all 30 algorithm runs for the three variables were 48 min, 4.38 min and 18.06 mg/L. For the 29 successful medium sized network runs the start time was 06:50, end time 07:40 and chemical concentration of 837 mg/L and the root mean square errors were 6.06 min, 12.36 min and 299.84 mg/L. The algorithmic framework successfully narrows down the potential sources of contamination leading to a pollution source identification, start and ending time of the event and the contaminant chemical concentration.

## 1. Introduction

Identifying the source of contamination in a water supply network is an important task since a contamination event is potentially hazardous to the human population in an urban environment. Additionally, a fast identification of a pollution source enables the governing authorities to rapidly react in order to stop the further spread of the contaminant through the water supply network.

Researches have tackled the issue of water supply networks contamination in several ways which include an optimal positioning of water quality sensors in a network [1,2,3] to facilitate the source identification process and optimally cover all possible intrusion points, rapid contamination event response procedures [4,5,6] and simulation-optimization methods for contamination source detection and duration based on simulation of the water network contamination event [7,8,9]. Many researches have incorporated additional uncertainties into the hydraulic simulation process which include uncertain sensor measurements and water demand variability [10,11,12]. A recent and thorough review of various approaches in tackling the water supply pollution source identification problem can be found in Adedoja et al. [13].

The optimization-simulation approach for finding the source of pollution in a network entails that an optimization algorithm is being coupled with a water supply network hydraulic simulator and the difference between the measured sensor data and the optimization algorithm generated and simulated values (sensor water quality readings through a certain time interval) is being minimized. Through this procedure, the source of contamination, starting time and the end time of the contamination event and the contaminant chemical concentration are obtained.

Probabilistic approaches are also possible for determining the source of contamination in a water supply network and the methods for this approach that were used in previous studies include the Bayesian Belief Networks (BBN) [14,15,16] and backward probabilistic models [17] which show the ability to predict the source of the contamination with a high probability. Data-driven methods are also a possible tool for the water supply network contamination problem. In [18,19], a database was compiled by massive data mining of hydraulic and water quality simulation contamination events for a fast identification of a source in case of a real event. The sensor readings in case of a contamination event are matched with the simulation events from a database and in [19] a statistical maximum likelihood approach was used for matching. In [20], Monte Carlo (MC) water quality and hydraulic simulations were run in parallel and then used to detect the source of pollution with a certain criteria.

In [21] the logistic regression approach was used to determine top candidates for being the true contamination source with them additionally being explored with local search methods to determine other relevant variables of a contamination event (start time, end time and chemical concentration). The input data for the logistic regression were the sensor readings through time that were constantly updated with new data.

Data-driven models would include using machine learning algorithms to localize the contamination sources in a water supply network. Kim et al. [22] used an Artificial Neural Network (ANN) to find the source of pollution in a small network and Rutkowski and Prokopiuk [23] used a learning vector quantization Neural Network (LVQNN) to locate a zone with a supply network where a potential source of contamination would be located. Wang et al. [24] used Least Squares Support Vector Machines (LS-SVM) to enhance the reliability and accuracy of water sensor contamination detection.

Previous studies have extensively explored Monte-Carlo based methods in air pollution and groundwater pollution source detection problems. In Guo et al. [25] a Markov Chain Monte Carlo (MCMC) sampling method coupled with a Bayesian probabilistic approach was applied to find a source of unsteady atmospheric dispersion which was numerically modeled. Wade and Senocak [26] used Bayesian inference MCMC for the purpose of reconstructing multiple air pollution sources. The study was tested on real-field data and the method includes a ranking of the most probable number of pollution sources which is based on error analysis. In the work by Bashi-Azghadi et al. [27] Probabilistic Support Vector Machines (PSVM) and Probabilistic Neural Networks (PNN) were used to determine an unknown source of pollution in a groundwater system. Vesselinov et al. [28] studied the application of semi-supervised machine learning methods with synthetic and real measured data to identify contamination sources of chemical mixtures in groundwater flows.

In this study we present an algorithm which utilizes the ANN for classification of contamination source in a water supply network and Random Forests (RF) for prediction of contamination start time, end time and chemical concentration. The algorithm is built in a high performance computing (HPC) environment and uses a parallel tournament style selection of most probable contamination source node between a group of nodes. All network nodes are divided in chosen number of tournament groups, where each group is assigned to a single processing core. Monte Carlo (MC) hydraulic and water quality simulations using EPANET2 (Rossman [29]) are run in parallel and the obtained simulation results are used to create models for every tournament group. Network nodes are randomly distributed into tournament groups and the parameters for every simulation (contamination start time, end time and chemical concentration) are randomized. Each network node in a tournament group obtains the same number of results (or MC simulations). Once a node is selected as a winner in the tournament process from existing groups, new tournament groups are created with a reduced number of suspect nodes and this process is repeated until a stopping criterion is satisfied which in this case is the number of set tournament loops which is also a parameter of the algorithm. The whole algorithmic framework was built with a combination of the Python 3.7 programming language and Simple Linux Utility for Resource Management (SLURM) for HPC systems. The ANN classification and the RF regression analysis were done using the Python machine learning library scikit-learn 0.22. The algorithm was tested on two benchmark networks taken from previous studies and it shows good results but also a possibility for improvement with future studies. The algorithm shows a great ability to include the true source node as a top candidate among the remaining source nodes and is good at predicting the other relevant contamination event parameters which can be further used for coupling with optimization algorithms.

## 2. Materials and Methods

### 2.1. Water Supply Network Benchmarks

The algorithmic framework was tested on two benchmark networks—the Net3 example from EPANET2 and the Richmond water supply network (Van Zyl [30]).

**Figure 1:**
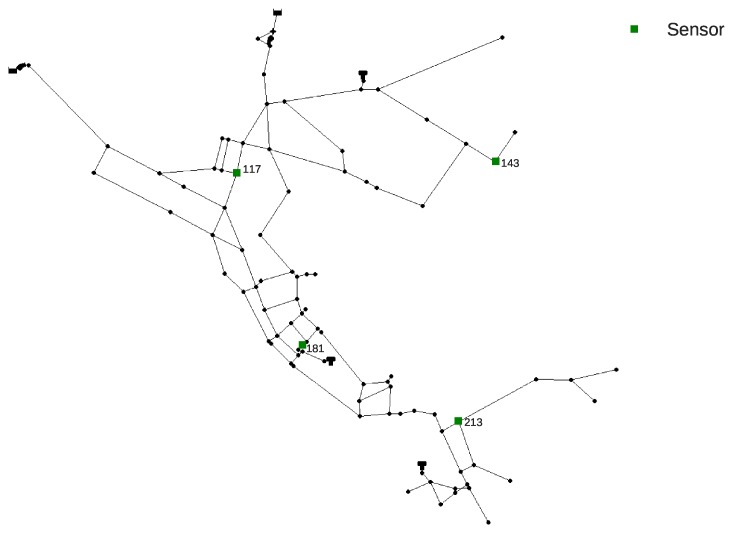
Net3 water supply network layout with sensor positioning by Preis and Ostfeld [7].

The Net3 EPANET2 benchmark water supply network consists of 92 nodes and was specifically made for water quality hydraulic simulations. The simulation parameters are set as: the total simulation time is 24 h with a 10 min hydraulic time step and 5 min water quality time step and a 10 min pattern time step. The already optimized water quality sensor layout was set as the one from the work by [7] (network nodes 117, 143, 181 and 213 are set as sensors). The Net3 network layout with the sensor placement can be seen in Figure 1. In each MC simulation of Net3, both the start and the end times ($S_{m}$, $E_{m}$) of the contamination event were randomly set from 0 to 24 h with an obvious restriction that $E_{m}>S_{m}$. The value of $C_{m}$ was randomly chosen from an interval from 10 to 1000 mg/L and was kept constant throughout the whole contamination scenario. The sensors used in Net3 recorded data during the whole 0–24 h interval for every hour (a total of 25 water quality measurements per sensor) which means that there were 100 input features for the RF regression analysis.

**Figure 2:**
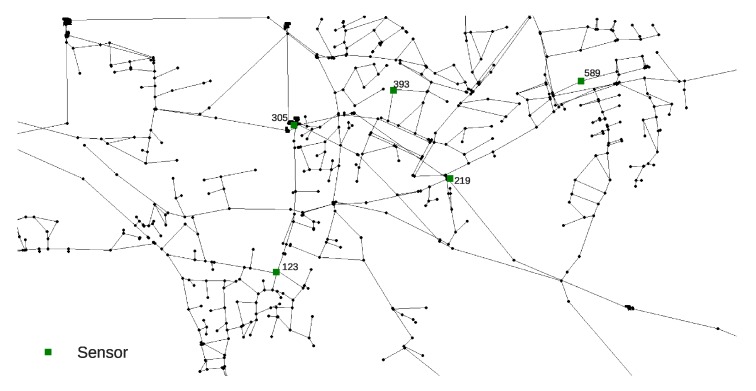
Richmond water supply network layout with sensor positioning by Preis and Ostfeld [7].

The Richmond network consists of 865 nodes and it was obtained from The Centre for Water Systems (CWS) at the University of Exeter [31]. Simulation time was set as 72 h with a 1 h hydraulic time step, a 5 water quality time step and a 1 pattern time step. The sensor layout was set according to the work by Preis and Ostfeld [7] (nodes 123, 219, 305, 393 and 589 are sensors nodes). The Richmond water supply network layout detail can be seen in Figure 2. The selection of random parameters for each MC Richmond network simulation were defined the same way as for the Net3 network. The Richmond network sensors recorded data during the 0–72 h interval for every hour (a total of 73 measurements per sensor) making a total of 365 input features for the RF regression analysis.

### 2.2. Algorithmic Framework

**Figure 3:**
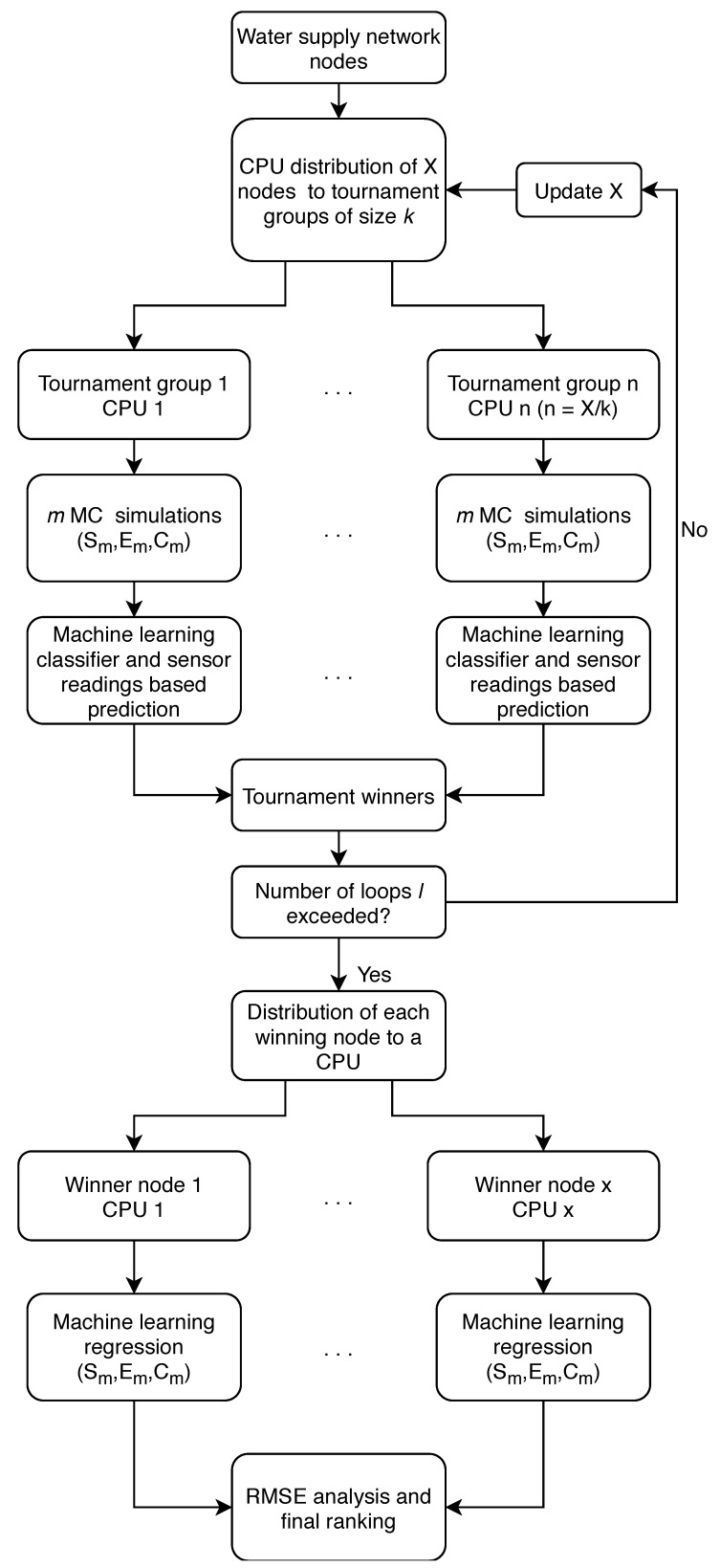
Algorithm flowchart.

The algorithm presented in this study was designed to work in a HPC environment in order to detect the water supply network contamination source in a rapid and efficient way. It is based on distributing all potential contamination source nodes of a water supply network into subgroups for which MC simulations would be done in conjunction with machine learning methods to determine which node would best fit to be the true source node of contamination. In this way, each node in a subgroup would be a part of a tournament in which the node with the highest probability of being the contamination source would continue to the next tournament round. After each tournament, the winning nodes would be redistributed in a new tournament group until a predetermined number of tournaments was reached. Each CPU in a HPC system is assigned a tournament node group and after each tournament the total number of used CPUs would decrease since the losing nodes would be discarded and the remaining nodes which are fewer would form new tournament groups. The flowchart of the whole algorithmic framework is shown in Figure 3.

As seen in the flowchart, the algorithm is initialized with reading all potential source nodes X in a water supply network and then distributing them in tournament groups of constant size k which is a parameter of the algorithm and can be freely selected. As each tournament group is assigned to a CPU, the number of used CPUs n is determined with n = X/k. After distributing the tournament groups to each CPU, MC simulations are performed m times (m/k times for each suspect node in the tournament group with ideally the modulus of m/k being zero—if not, a node is randomly selected for the additional run) with randomly selected starting $S_{m}$ and ending $E_{m}$ times of the contamination event, and the contaminant chemical concentration $C_{m}$. Input and output data of each MC simulation are being saved for each suspect node x in every tournament group n.

After the MC simulations are done, the input (sensor readings through time) and output data (source node with used $S_{m}$, $E_{m}$ and $C_{m}$) are used for training each tournament group’s machine learning (ML) model. The ML model used can be any ML classifying algorithm which supports the prediction of multiple classes. ML output variable set consists of all network nodes that are within that tournament group. After the model was trained with the MC generated data, the sensor readings of the contamination event are being used for the ML prediction of the most probable source node in each tournament group. The nodes with the highest probability in all tournament groups are considered to be the tournament winners. After every used CPU generated a tournament winner, a list of all winners is compiled and if the number of set tournament loops l is not exceeded, the tournament process and distribution is repeated and the number of nodes X is updated (it is equal to the number of winners). In this case, the number of X should be smaller than in the previous tournament loop and consequently the number of used CPUs is reduced since it is dependant on the number of nodes for distribution. It is important to note that each winning node’s input and output data is saved from every tournament loop.

If the freely selected algorithm parameter l is exceeded, each winning node is then assigned again to a CPU and with its previously obtained MC input and output data, a ML model is trained and a prediction is performed for the remaining variables of the contamination event ($S_{m}$, $E_{m}$ and $C_{m}$). The predicted values of $S_{m}$, $E_{m}$ and $C_{m}$ of each winning node’s ML regression model are then used for simulating the contamination event scenario and the obtained sensor readings are then compared with the real contamination event sensor readings with a RMSE analysis, which in turn creates a ranking where the node with the smallest RMSE is placed at the top.

This whole algorithmic framework was built within the open source workload manager for cluster systems SLURM and the Python 3.7 programming language.

### 2.3. ANN Classifier

In the previous sub section, the tournament ML classifier was generally defined in the whole algorithmic framework and basically any ML algorithm which can predict multiple classes can be used. In this study, the ANN algorithm is used for classifying the most probable source nodes in a tournament group.

The Multi-layer Perceptron (MLP) type of ANN was used from the Python 3.7 machine learning library scikit-learn 0.22 [32]. The MLP ANN was constructed with both input and output layers and three hidden layers in between. Both first and last hidden layers consisted of 100 neurons, while the middle hidden layer was formed with 500 neurons. The stochastic gradient-based optimizer ADAM for MLP weights optimization was selected through the process of hyper parameter tuning, just as the number of neurons in every ANN layer.

With a preliminary analysis of the possible input variables of the ANN MLP model it was determined that great accuracy of the model can be achieved if only the maximum values of the chemical concentration recorded per sensors through a time interval in the water supply network are used. The preliminary analysis was done through 10 runs and each run when the true source node was in the top 6 of the final nodes was considered successful. The analysis was done on the Net3 benchmark network with the contaminant source node 119 as described in Section 3.1. The true source node was a part of the top 6 ranking suspect nodes for all of the 10 preliminary runs.

This means that the number of neurons at the input layer is equal to the number of sensors used in the water supply network. Furthermore, using the whole time interval of all sensor water quality readings as ANN MLP inputs was tested in the preliminary analysis and it was found that the performance was not better (8 out of 10 runs were successful) than using the maximum values of recorded water quality through time, so naturally, the maximum values per sensor were used as inputs since the number of ML model features is much smaller that way.

The output of the MLP was a list of all tournament group nodes and an assigned probability of each node being the true contamination source after the real contamination event sensor reading was evaluated with the trained model.

**Figure 4:**
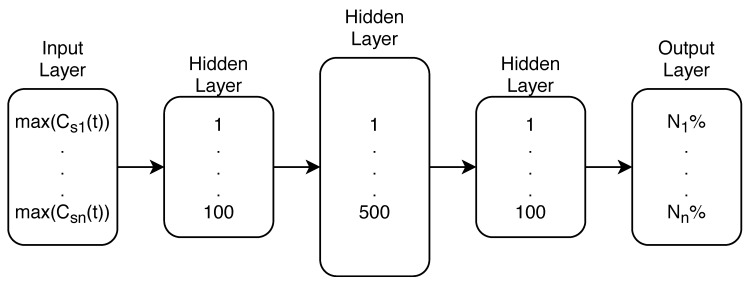
MLP with all layers.

In Figure 4 the whole MLP can be seen with $max(C_{s}n\left(t\right))$ being the maximum concentration recorded by the n-th sensor in the network and $N_{n}\%$ being the probability that the tournament node n is the true source node. All of the MC generated input and output data (of each tournament group) is used to train the ANN model for each tournament group as the goal of the classifier is to predict the most probable contamination source node of each tournament group. The success was assessed by observing the prediction of the whole algorithmic process and not the accuracy of each tournament group ANN model.

### 2.4. RF Regression

The ML regression model for each tournament winning node after the parameter l was exceeded in the algorithmic framework can also be done with any ML algorithm which supports multi output regression. The RF algorithm (Breiman [33]) from scikit-learn 0.22 was selected for this purpose. All parameter values of the algorithm were set as default except for the number of estimators (trees in the random forest) which was set to be 200 with the process of hyperparameter optimization.

**Figure 5:**
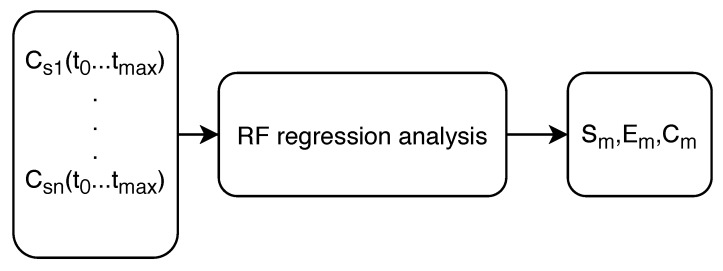
Flowchart of the RF regression analysis.

The input values for the RF regression analysis were sensor water quality readings throughout the whole time interval of the simulation (unlike the inputs used for the MLP ANN) and the output variables were the predicted values of $S_{m}$, $E_{m}$ and $C_{m}$ for every winner node. A flowchart of the RF regression is seen in Figure 5 with $C_{sn}\left(t_{0}\ldots t_{max}\right)$ representing the chemical concentration recorded by the sensor n during simulation time.

## 3. Results and Discussion

### 3.1. Net3 Network Contamination Scenario

**Figure 6:**
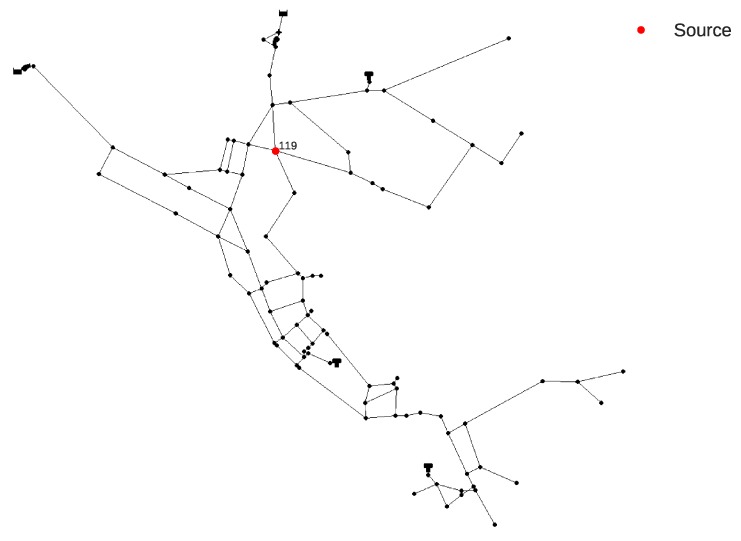
Net3 water supply network contamination source location.

The contamination event scenario for the Net3 benchmark network was chosen to be from the same node (119) as in the one from the work by Preis and Ostfeld [7] and the location can be seen in Figure 6. The contamination event characteristics at source node 119 were freely chosen with the event starting at 14:20 h and lasting until 20:20 h with a constant chemical mass inflow of 813.7 mg/L.

The selected number of algorithm loops l was 3, the number of m (MC simulations for every tournament group) was 200 and the size of a tournament group k was 2, which means that with 92 initial water supply network nodes, the number of used CPUs for every tournament group was 46 and after every loop that number was halved. After three loops, the number of tournament winners was 11, which means that 11 CPUs were used for the RF regression analysis and prediction of other relevant variables.

The contamination source search was repeated 30 times since there the algorithm consists of a stochastic component (MC simulations). In 22 out of 30 runs the true source node was the suspect node with the highest probability and in the remaining eight runs the true source node was a part of the final winners list, which means that the ANN classification can successfully narrow down the search space from 92 to 11 nodes in this case. The average run which includes MC simulations, ANN classification and RF regression lasted for 8 min (even though the RF regression lasted only for 8 s). The algorithm was run (at its initial loop) on 46 Intel Xeon E5 CPUs (two cluster nodes). Out of the other eight runs when the true source node was not ranked first, it was always in the top six of the tournament winners.

**Figure 7:**
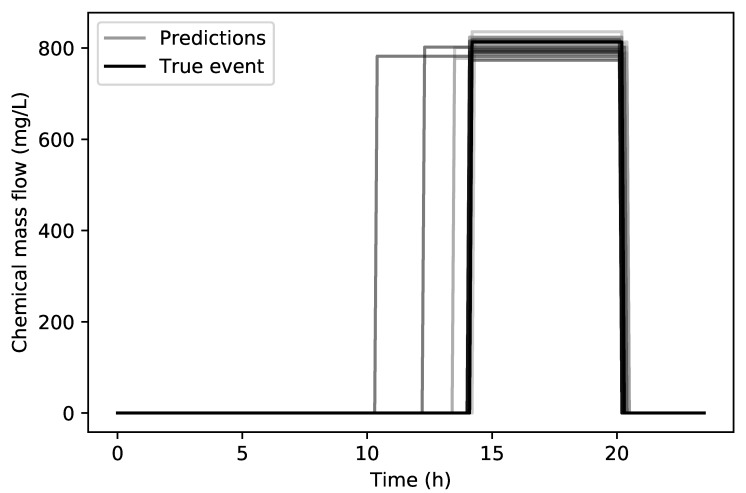
Net3 network true contamination event (black) and predicted contamination events (grey).

In Figure 7 a comparison can be seen between the true contamination event (14:20 h to 20:20 h with 813.7 mg/L) and all of the 30 predicted contamination events for the true source node. It can be seen that the end time prediction of the event is very accurate while the starting time only lacking in accuracy on three runs. The overall RMSE for the starting time for all 30 runs is 48 min, the end time is 4.38 min and the chemical concentration is 18.06 mg/L. The average RMSE For the three of the worst runs with respect to the starting time was 2.47 h. In Table 1, a summary of all runs can be seen through the RMSE analysis and the successful runs represent how many times of the total of 30 runs the true source node was part of the final tournament. The minimum and maximum errors for $S_{m}$, $E_{m}$ and $C_{m}$ for all 30 runs are presented in Table 2.

In Table 3 the best and worst runs are compared with the true contamination event parameters for $S_{m}$, $E_{m}$ and $C_{m}$. The overall best and worst runs are calculated (individual RMSE) by taking into account all of the three variables.

**Figure 8:**
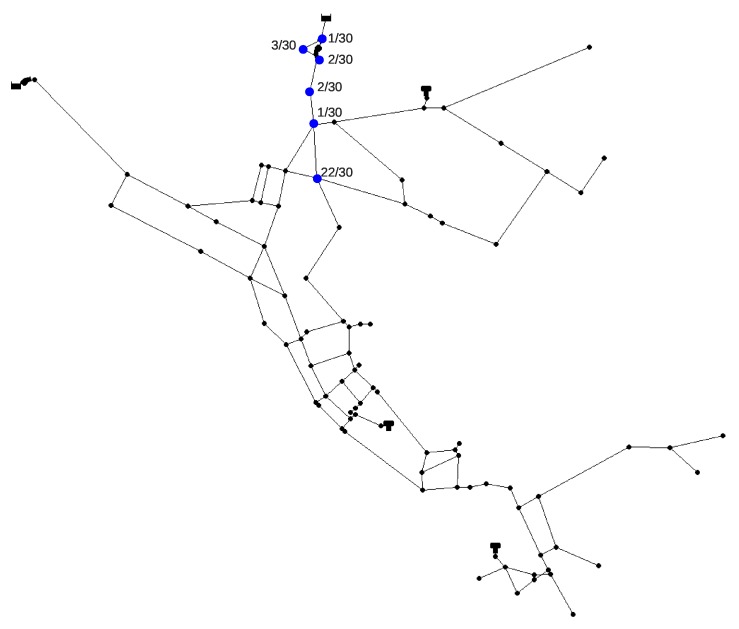
Number of times out of 30 runs for which a node (marked blue) was ranked as first for the Net3 contamination event.

In Figure 8 the nodes which were ranked first in the 30 runs can be seen along with a corresponding number of times they were ranked first. It can be observed that the nodes are topologically clustered together. This is expected since due to the multimodal nature of the problem.

### 3.2. Richmond Network Contamination Scenario

**Figure 9:**
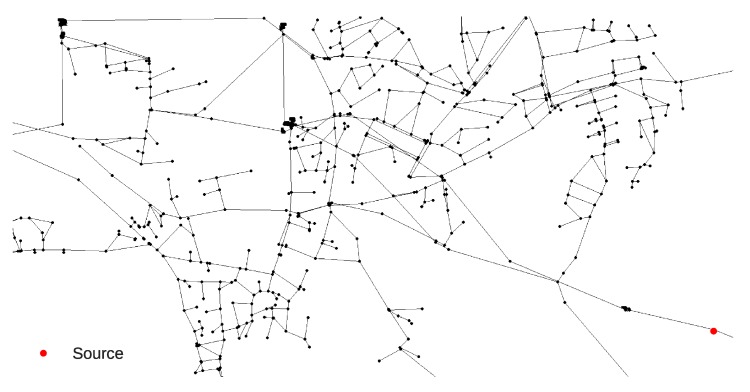
Richmond water supply network contamination source location.

The Richmond network contamination event scenario was chosen to start at the same node (153) as in the work by Preis and Ostfeld [7] and the location can be seen in Figure 9. The contamination event characteristics at source node 153 were chosen with the event starting at 06:50 h and lasting until 07:40 h with a constant chemical mass inflow of 837 mg/L.

The selected number of algorithm loops l was 1, the number of m (MC simulations for every tournament group) was 2500 and the size of a tournament group k was 4, which means that with 865 initial water supply network nodes, the number of used CPUs for every tournament group was 432. After 1 loop the number of tournament winners was 217, which means that 217 CPUs were used for the RF regression analysis and prediction of other relevant variables.

The contamination source search was repeated 30 times just like for the Net3 contamination search. The true source node was ranked first in seven out of 30 runs and it was in the tournament winners list 29 out of 30 times which means that the true source node was not subjected to the RF regression analysis for only one run. The total average run time of the MC simulations and the ANN classification was 41 min and 75 s for the RF regression analysis. The algorithm was run (at its initial loop) on 432 Intel Xeon E5 CPUs. Even though it was ranked first in only seven runs, that was the most number of times a node was ranked first out of the 30 runs. In the 29/30 runs it was in the winners list (which consisted of 217 nodes) it always finished in the top 10 after RF regression was completed.

**Figure 10:**
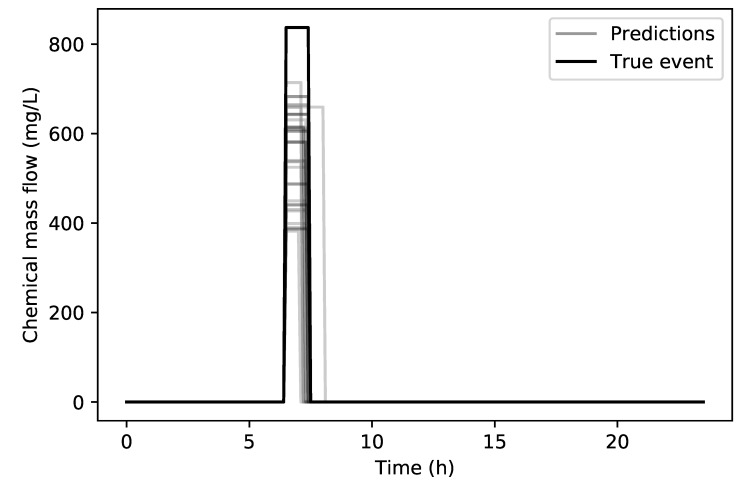
Richmond network true contamination event (black) and predicted contamination events (grey).

In Figure 10 the comparison between the true contamination event and the predicted contamination events can be seen. The starting and ending times RMSE for the 29 of the 30 total runs are 6.06 min and 12.36 min respectively, while the chemical concentration RMSE is 299.84 mg/L. The starting and ending times of the predictions are in good agreement with the true values but the chemical concentration value is underestimated by the RF regression.

A RMSE analysis summary of all runs with the number of successful runs can be seen in Table 4. The minimum and maximum errors for all 29 runs for the Richmond network are shown in Table 5 and in Table 6, the best and worst runs comparison is shown in terms of all the predicted variables ($S_{m}$, $E_{m}$ and $C_{m}$).

### 3.3. Algorithm Parameters Investigation

In this subsection, an analysis of the influence of algorithm parameters is given. The required number of MC simulations m for each node, the tournament group size k and the number of algorithm loops l are separately explored. The examination of m, k and l was done on the Net3 water supply network with the previously defined contamination event scenario (source at node 119, $S_{m}$ = 14:20 h, $E_{m}$ = 20:20 h and $C_{m}$ = 813.7 mg/L). Each different setup (of m, k and l) was independently run 10 times and a run was considered successful if the node was present in the final ranking after the RF regression and RMSE analysis.

Firstly, the influence of parameter m on the prediction accuracy was explored and a complete summary can be seen in Table 7. The selected tournament group size k for all runs was 2 and the number of loops l was set as 1 during the exploration of parameter m.

The first column of Table 7 represents the total number of MC simulations per tournament group, which means that since the tournament group size was 2 for each run, each node in the tournament group was the source node in m/2 MC simulations. When observing the analysis of the parameter m in Table 7 it can be seen that the higher the value of the parameter is, both contamination event prediction accuracy (as seen through the decrease $S_{m}$, $E_{m}$ and $C_{m}$ RMSE) and the average computation time per run (last column) increases. This is expected since more randomly generated data covers more possible scenarios and more input data for the ML model enables a wider and more accurate solution space exploration. Additionally, the best and worst ranks of the true source node are shown and the number of times the true source node won (Times won meaning the rank was 1).

When m was set as 80 the number of successful runs was 10 and the worst possible rank was 6. This means that after 2 min of computation time on average, the search space was reduced from 92 total nodes to 6, which is a reduction of 93.5%. The set of runs when m was 400 could be considered as the first set of runs when the results are acceptable in terms of finding the true source node since it was the winner 50% of the time.

When the value of m is 2000 and above it can be seen that there is not a significant change in prediction accuracy as the RMSE values of $S_{m}$ and $C_{m}$ exhibit stability and minor oscillations, while $E_{m}$ has showed a steady convergence to the same value as the true contamination scenario for all 10 runs.

For further exploration of the tournament group size k, the chosen m was 800 as it exhibited a reasonable computation run time, accuracy in terms of the average RMSE and the number of times the true source node was ranked first. The same scenario was chosen as the one for the investigation of m with the number of tournament loops l set as 1. Five different tournament group sizes k were explored and are summarized in Table 8.

From the results presented in Table 8 it can be observed that when the tournament group is larger, both accuracy and prediction reliability decrease. Furthermore, besides tournament group sizes of 2 and 4, a reasonable result in terms of reliability is achieved with k = 10 with a total search space reduction of 94.6% and even a 50% winning rate in the 10 successful runs. The number of used CPUs for each tournament group size is added and with the given as the last column of the Table 8. Even though a tournament group size of 2 is not that impressive when compared to those of 4 and 10 in the categories of best and worst rank and times won, the achieved overall $S_{m}$, $E_{m}$ and $C_{m}$ RMSE shows that it is undoubtedly more accurate.

Lastly, the influence of the number of tournament loops l is investigated and a summary of the results is shown in Table 9. The same scenario was used as for the exploration of previous two parameters with k = 2 and with a total number of MC simulations m = 800. An additional column m/L was added to the Table 9 which defines the number of MC simulations m (of a tournament group) per every loop l.

It can be observed that increasing the number of loops l up to a certain value increases the accuracy and reliability of the algorithm. Even though the total number of MC simulations is the same for every run and the computational strain in that sense is similar, adding more loops decreases the number of used CPUs after every tournament loop since losing nodes are omitted and that can be considered as a great advantage.

The value of $S_{m}$, $E_{m}$ and $C_{m}$ RMSE does not differ much for all tested loops l since the total number of MC simulations is preserved. When the number of loops is set to 8, the successful number of runs dropped as the number of MC simulations per loop (m/L) was not high enough and the true source node was not in the final RF analysis ranking for one run. This was also observed for smaller values of m in Table 7. It can be argued that a higher number of loops positively affects the success of the algorithm in predicting the relevant variables (source node, $S_{m}$, $E_{m}$ and $C_{m}$) since there is a higher chance that a main tournament top ranking rival to the true source node is omitted in the process of removing losing nodes after every tournament loop. However, setting l too high could result in unsuccessful runs as well (due to a small m/L) as it can be seen in Table 9.

## 4. Conclusions

In this paper a novel algorithmic framework for water supply network contamination source node identification is presented and tested on two different benchmark networks. The algorithm is specifically created for massively parallel HPC systems and it utilizes a combination of MC simulations and ML methods to identify the contamination event source node and all the relevant variables such as starting and ending times of the event and the contaminant chemical concentration.

The algorithm is based on running an equal number of MC simulations on a group of nodes in parallel and then selecting the node (via MLP ANN) with the highest probability of being the true contamination source node as the winner of the group (tournament). The number of MC simulations per tournament group is set as a parameter of the algorithm just like the number of tournament loops and the size of a tournament group. After a set of tournament winners is created, the algorithm utilizes a ML regression analysis using RF in parallel and creates a ranking of potential source nodes based on a simple error analysis with the true contamination event sensor data recorded.

The novel algorithmic framework, tested on two realistic and complex benchmark networks cases, displays the capability to narrow down the search space for the source node efficiently, leading to a pollution source identification. The algorithm can be also used in predicting the starting and ending times of the contamination event and the contaminant chemical concentration.

An investigation of algorithm’s parameters which are the number of MC simulations, size of a tournament group and number of loops was also conducted. It is demonstrated that increasing the number of MC simulations is beneficial to the algorithm’s ability to predict the true source node and relevant variables since more randomly generated data entails a broader solution space coverage, however this comes with an increase in computational time. It was observed that in order to increase the reliability of an accurate prediction the size of a tournament group should be as low as possible, depending on computational resources. Increasing the number of tournament loops shows to be advantageous in prediction accuracy; however, the number of MC simulations for each tournament loop should be high enough in order to preserve the reliability of prediction.

Further research is needed in determining the connection between the network size (initial number of potential source nodes) and the number of MC simulations needed to cover the search space of the contamination event efficiently and thoroughly. Furthermore, additional research should be done regarding the used ML methods investigating the use of different classifiers for the tournament group winner selection and various ML algorithms capable of multi-output regression analysis for the final node ranking. It would be also possible to couple the algorithm with simulation-optimization methods for an even faster convergence towards a true pollution source node detection in a way that only the tournament winners are subjected to the optimization process.

## Figures and Tables

**Table 1:** Contamination event RMSE analysis for Net3 network of all 30 runs.

Successful Runs	$S_{m}$	$S_{m}$ RMSE	$E_{m}$	$E_{m}$ RMSE	$C_{m}$	$C_{m}$ RMSE
30	14:20 h	48 min	20:20 h	4.38 min	813.7 mg/L	18.06 mg/L

**Table 2:** Net3 network minimum and maximum errors ($S_{m}$, $E_{m}$ and $C_{m}$) for 30 runs.

min $S_{m}$	max $S_{m}$	min $E_{m}$	max $E_{m}$	min $C_{m}$	max $C_{m}$
−3.8 h	+0.2 h	0.0 h	+0.2 h	−0.31 mg/L	−40.11 mg/L

**Table 3:** Net3 network contamination event results comparison between true, best and worst of the total 30 runs.

Run	$S_{m}$	$E_{m}$	$C_{m}$
True	14:20 h	20:20 h	813.7 mg/L
Best	14:20 h	20:20 h	813.4 mg/L
Worst	14:10 h	20:20 h	773.6 mg/L

**Table 4:** Contamination event RMSE analysis for Richmond network of all 30 runs.

Successful Runs	$S_{m}$	$S_{m}$ RMSE	$E_{m}$	$E_{m}$ RMSE	$C_{m}$	$C_{m}$ RMSE
29	06:50 h	6.06 min	07:40 h	12.36 min	837 mg/L	299.84 mg/L

**Table 5:** Richmond network minimum and maximum errors ($S_{m}$, $E_{m}$ and $C_{m}$) for 30 runs.

min $S_{m}$	max $S_{m}$	min $E_{m}$	max $E_{m}$	min $C_{m}$	max $C_{m}$
0.0 h	+0.4 h	−0.4 h	+0.6 h	−122.81 mg/L	−446.81 mg/L

**Table 6:** Richmond network contamination event results comparison between true, best and worst of the total 30 runs.

Run	$S_{m}$	$E_{m}$	$C_{m}$
True	06:50 h	07:40 h	837 mg/L
Best	06:50 h	07:40 h	714.2 mg/L
Worst	06:50 h	08:00 h	390.2 mg/L

**Table 7:** Influence of the number of MC simulations on the accuracy and efficiency of the algorithm.

m	Successful Runs	Best Rank	Worst Rank	Times Won	$S_{m}$ RMSE	$E_{m}$ RMSE	$C_{m}$ RMSE	Average Run Time
20	9	1	7	2	1.44 h	1.21 h	291.89 mg/L	50 s
40	9	3	8	0	1.76 h	1.11 h	371.93 mg/L	70 s
80	10	1	6	1	0.75 h	0.36 h	283.45 mg/L	120 s
100	10	1	6	4	0.18 h	0.21 h	187.39 mg/L	160 s
200	10	1	6	4	0.58 h	0.18 h	109.40 mg/L	270 s
400	10	1	6	5	0.83 h	0.10 h	39.23 mg/L	420 s
800	10	1	5	6	0.14 h	0.11 h	9.43 mg/L	560 s
1000	10	1	6	9	0.32 h	0.03 h	15.69 mg/L	820 s
1200	10	1	1	10	0.06 h	0.00 h	11.61 mg/L	980 s
2000	10	1	1	10	0.03 h	0.00 h	3.57 mg/L	1400 s
3000	10	1	1	10	0.03 h	0.00 h	4.46 mg/L	2100 s
4000	10	1	1	10	0.00 h	0.00 h	4.09 mg/L	3200 s
5000	10	1	1	10	0.03 h	0.00 h	1.41 mg/L	4000 s
6000	10	1	1	10	0.03 h	0.00 h	3.59 mg/L	4800 s
10,000	10	1	1	10	0.00 h	0.00 h	2.00 mg/L	6300 s

**Table 8:** Influence of the number of the tournament group size k on the accuracy and efficiency of the algorithm.

k	Successful Runs	Best Rank	Worst Rank	Times Won	$S_{m}$ RMSE	$E_{m}$ RMSE	$C_{m}$ RMSE	CPUs Used
2	10	1	5	6	0.14 h	0.11 h	9.43 mg/L	46
4	10	1	5	7	0.27 h	0.08 h	27.46 mg/L	23
10	10	1	5	5	0.74 h	0.17 h	83.16 mg/L	9
40	5	1	3	1	0.35 h	2.19 h	215.59 mg/L	2
80	4	1	2	2	0.27 h	0.53 h	143.04 mg/L	2

**Table 9:** Influence of the number of the tournament loops l on the accuracy and efficiency of the algorithm.

l	Successful Runs	Best Rank	Worst Rank	Times Won	$S_{m}$ RMSE	$E_{m}$ RMSE	$C_{m}$ RMSE	m/L
1	10	1	5	6	0.14 h	0.11 h	9.43 mg/L	800
2	10	1	6	8	0.18 h	0.08 h	23.20 mg/L	400
3	10	1	4	8	0.11 h	0.04 h	15.42 mg/L	267
4	10	1	3	9	0.08 h	0.03 h	14.71 mg/L	200
5	10	1	3	7	0.07 h	0.08 h	14.57 mg/L	160
6	10	1	2	8	0.07 h	0.05 h	23.29 mg/L	134
7	10	1	1	10	0.03 h	0.05 h	14.77 mg/L	115
8	9	1	3	7	0.07 h	0.07 h	25.49 mg/L	100

## Abbreviations

The following abbreviations are used in this manuscript:

BBN	Bayesian Belief Networks
MC	Monte Carlo
ANN	Artificial Neural Network
LVQNN	Learning Vector Quantization Neural Network
LS-SVM	Least Squares Support Vector Machines
MCMC	Markov Chain Monte Carlo
PSVM	Probabilistic Support Vector Machines
PNN	Probabilistic Neural Networks
RF	Random Forests
HPC	High Performance Computing
CWS	Center for Water Systems
CPU	Central Processing Unit
ML	Machine Learning
SLURM	Simple Linux Utility for Resource Management
RMSE	Root Mean Square Error
MLP	Multi-layer Perceptron
